# Supplementary material for: Bayesian refinement of protein structures and ensembles against SAXS data using molecular dynamics
Source: PLoS Comput Biol. 2017 Oct 18;13(10):e1005800. doi: 10.1371/journal.pcbi.1005800 (PMC5662244; doi:10.1371/journal.pcbi.1005800)
Supplement: S1 Archive — (BZ2) [file pcbi.1005800.s011.tar.bz2 › gromacs_4.62_bayesian_saxs_refi/share/html/online/g_sorient.html]

g\_sorient


| g\_sorient Main Table of Contents |

**VERSION 4.6  
Sat 19 Jan 2013**

---

### Description

g\_sorient analyzes solvent orientation around solutes.
It calculates two angles between the vector from one or more
reference positions to the first atom of each solvent molecule:

θ\_1: the angle with the vector from the first atom of the solvent
molecule to the midpoint between atoms 2 and 3.  
θ\_2: the angle with the normal of the solvent plane, defined by the
same three atoms, or, when the option -v23 is set,
the angle with the vector between atoms 2 and 3.

The reference can be a set of atoms or
the center of mass of a set of atoms. The group of solvent atoms should
consist of 3 atoms per solvent molecule.
Only solvent molecules between -rmin and -rmax are
considered for -o and -no each frame.

-o: distribtion of cos(θ\_1) for rmin<=r<=rmax.

-no: distribution of cos(θ\_2) for rmin<=r<=rmax.

-ro:  and <3cos(^2θ\_2)-1> as a function of the
distance.

-co: the sum over all solvent molecules within distance r
of cos(θ\_1) and 3cos(^2(θ\_2)-1) as a function of r.

-rc: the distribution of the solvent molecules as a function of r

### Files

| option | filename | type | description |
| --- | --- | --- | --- |
| **-f** | traj.xtc | Input | Trajectory: xtc trr trj gro g96 pdb cpt |
| **-s** | topol.tpr | Input | Structure+mass(db): tpr tpb tpa gro g96 pdb |
| **-n** | index.ndx | Input, Opt. | Index file |
| **-o** | sori.xvg | Output | xvgr/xmgr file |
| **-no** | snor.xvg | Output | xvgr/xmgr file |
| **-ro** | sord.xvg | Output | xvgr/xmgr file |
| **-co** | scum.xvg | Output | xvgr/xmgr file |
| **-rc** | scount.xvg | Output | xvgr/xmgr file |

### Other options

| option | type | default | description |
| --- | --- | --- | --- |
| **-[no]h** | bool | no | Print help info and quit || **-[no]version** | bool | no | Print version info and quit || **-nice** | int | 19 | Set the nicelevel || **-b** | time | 0 | First frame (ps) to read from trajectory || **-e** | time | 0 | Last frame (ps) to read from trajectory || **-dt** | time | 0 | Only use frame when t MOD dt = first time (ps) || **-[no]w** | bool | no | View output .xvg, .xpm, .eps and .pdb files || **-xvg** | enum | xmgrace | xvg plot formatting: xmgrace, xmgr or none || **-[no]com** | bool | no | Use the center of mass as the reference postion || **-[no]v23** | bool | no | Use the vector between atoms 2 and 3 || **-rmin** | real | 0 | Minimum distance (nm) || **-rmax** | real | 0.5 | Maximum distance (nm) || **-cbin** | real | 0.02 | Binwidth for the cosine || **-rbin** | real | 0.02 | Binwidth for r (nm) || **-[no]pbc** | bool | no | Check PBC for the center of mass calculation. Only necessary when your reference group consists of several molecules. |

---

http://www.gromacs.org  
gromacs@gromacs.org
